# *Caenorhabditis elegans brc-1* mutation increases the number of COSA-1 foci in *him-8* and *zim-2* mutants

**DOI:** 10.17912/micropub.biology.001077

**Published:** 2024-07-25

**Authors:** Takamune T. Saito, Koki Yamamoto, Hirohito Minami, Taiki Tsujiue

**Affiliations:** 1 Department of Genetic Engineering, Faculty of Biology-Oriented Science and Technology, Kindai University, Kinokawa, Wakayama, Japan; 2 Graduate School of Biology-Oriented Science and Technology, Kindai University, Kinokawa, Wakayama, Japan; 3 Present address: Graduate School of Biostudies, Kyoto University, Kyoto, Japan

## Abstract

Crossover designation factors such as COSA-1 are concentrated at the specific DNA double-strand break (DSB) sites to promote crossover formation. *zim-1* mutants, which show defects in the homologous chromosome pairing of chromosomes II and III, increase the COSA-1 foci/normal bivalent state compared to the expected value. The excess designation was suppressed by an additional mutation in *brc-1* in *zim-1* mutants. We demonstrated that the number of COSA-1 foci in *him-8* and *zim-2* mutants, showing defects in the pairing of the X and V chromosomes, respectively, increased compared to the expected value, and *brc-1* mutation accelerated the number of COSA-1 foci in oogenesis.

**Figure 1. Deletion of BRC-1 increases the number of COSA-1 and RAD-51 foci at late pachytene in him-8 and zim-2 mutants:**
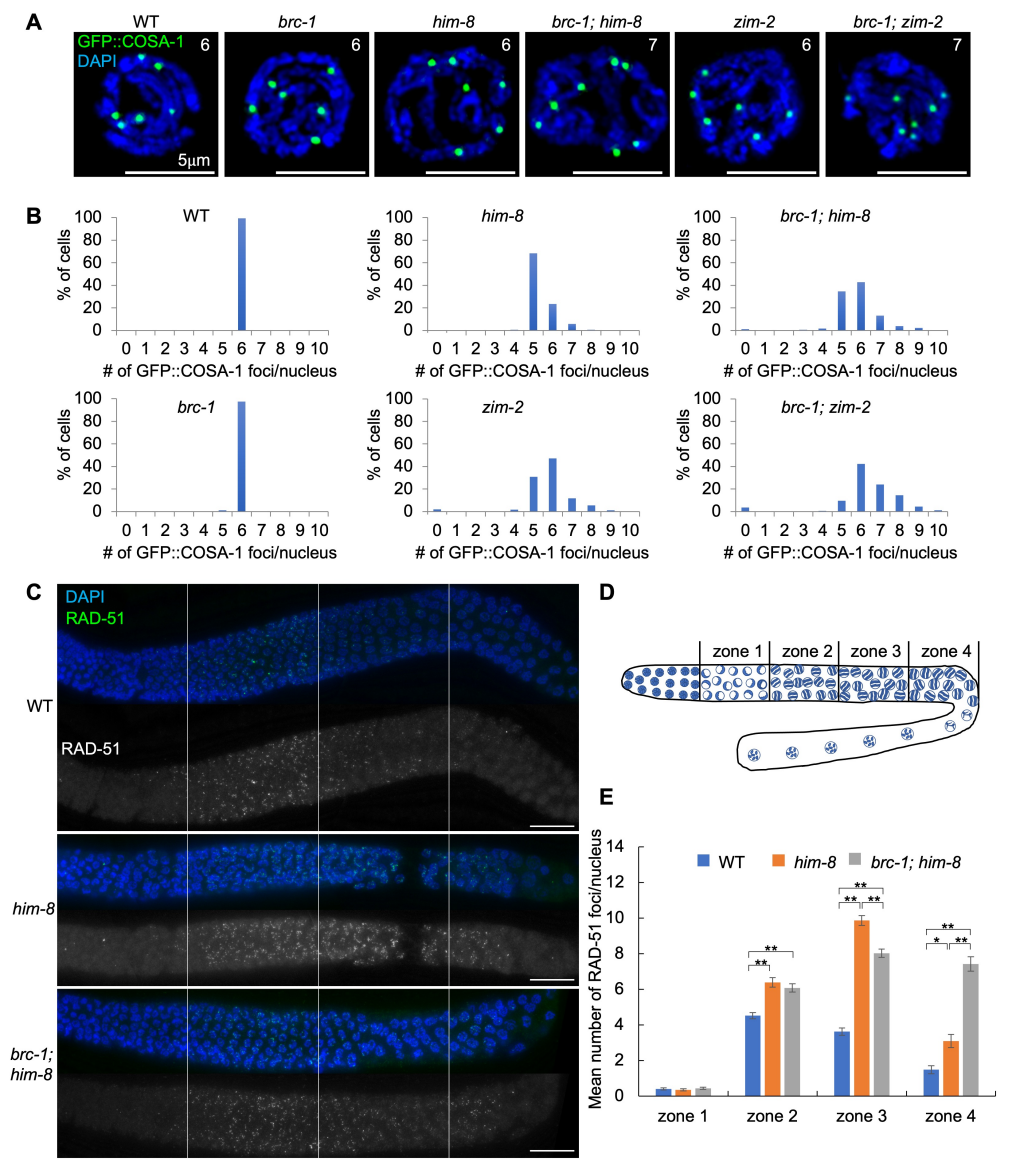
(A) Representative images of GFP::COSA-1 in indicated strains. The numbers in the panels indicate the number of GFP:: COSA-1 foci. Scale bars, 5µm. (B) Graphs showing the quantification of GFP::COSA-1 foci/nucleus in the late pachytene stage in each strain. N-value, WT = 6 gonads, 256 cells; *brc-1* mutants = 10 gonads, 267 cells; *him-8* mutants = 10 gonads, 272 cells; *brc-1 ; him-8* double mutants = 9 gonads, 182 cells; *zim-2* mutants = 18 gonads, 295 cells, *brc-1 ; zim-2* double mutants = 18 gonads, 326 cells. (C) Representative images of RAD-51 immunostaining in the gonads. Perpendicular white lines indicate the border of each zone. Scale bars, 20 µm. (D) Illustration of the gonad divided into four zones of equal length from the transition zone to late pachytene (zone 1, transition zone; zone 2, early pachytene; zone 3, mid-pachytene; and zone 4, late pachytene). (E) Graphs showing the average number of RAD-51 foci per nucleus in different zones. Error bars represent the standard error of the mean. Asterisks indicate statistical difference (*p=0.01732, **p<0.00001, two-tailed Mann-Whitney test). N-value, WT = 3 gonads, 250, 235, 138, and 90 cells per zone. *him-8* mutants = 3 gonads, 177, 206, 147, and 97 cells per zone. *brc-1 ; him-8* mutants = 4 gonads, 264, 224, 167, and 96 cells per zone.

## Description

Meiosis is a cell division process that generates haploid gametes from diploid parental cells. Crossover formation is essential for appropriate homologous chromosome segregation of meiosis I. A single crossover event per homologous chromosome pair is observed in wild-type *Caenorhabditis elegans* . The process of selecting a specific DNA double-strand break (DSB) as a future crossover site is called crossover designation (Kleckner *et al.* 2003; Gray and Cohen 2016). Crossover designation factors such as COSA-1 , MSH-5 , and ZHP-3 concentrate at the sites of DSBs, which are further repaired by crossover (Kelly *et al.* 2000; Jantsch *et al.* 2004; Bhalla *et al.* 2008; Yokoo *et al.* 2012). Therefore, six COSA-1 foci are observed in the nucleus at the late pachytene stage in the wild-type (Yokoo *et al.* 2012).

Homologous chromosomes begin to pair at the pairing center via pairing center-binding proteins ( ZIM-1 , ZIM-2 , ZIM-3 , and HIM-8 ) during the leptotene-zygotene transition (Phillips *et al.* 2005; Phillips and Dernburg 2006). The homologous pairing of chromosomes II and III is impaired in *zim-1* mutants (Phillips and Dernburg 2006) . Although *zim-1* mutants are expected to have four COSA-1 foci per nucleus, an average of 6.12 COSA-1 foci are observed in these mutants (Li *et al.* 2018). The mutation of *brc-1* , which is a homolog of human breast cancer gene 1 (BRCA1) (Futreal *et al.* 1994; Miki *et al.* 1994; Boulton *et al.* 2004), suppresses the increase in GFP::COSA-1 foci in *brc-1 ; zim-1* double mutants (4.3–4.8 COSA-1 foci/nucleus) (Li *et al.* 2018). In this study, we investigated whether *him-8* and *zim-2* mutants, in which the pairing of the X and V chromosomes, respectively, is defective (Phillips *et al.* 2005; Phillips and Dernburg 2006), show an increase in COSA-1 foci compared to the expected value (5 foci/nucleus), and whether the phenotypes were suppressed by the *brc-1* mutation.

We quantified the GFP::COSA-1 foci in the late pachytene stage in the wild-type, *him-8* , *brc-1 , zim-2* single mutants, and *brc-1 ; him-8* , *brc-1 ; zim-2* double mutants by 3D-fluorescent microscopy. As previously shown by many groups, six GFP::COSA-1 foci were observed in the wild-type ( Figure 1A, 1B). If a single crossover event was hypothesized to occur per homologous chromosome pair, then we would expect to observe five GFP::COSA-1 foci/nucleus in *him-8* mutants with unpaired X chromosomes and in *zim-2* mutants with unpaired chromosome V. However, in addition to five foci of GFP::COSA-1/nucleus, we observed that 30.5% and 65.4% of cells had more than six foci of GFP::COSA-1 in *him-8* and *zim-2* mutants, respectively ( Figure 1A, 1B). This phenotype was similar to that observed in *zim-1* mutants (Li *et al.* 2018). These data suggest that the interchromosomal effect causes excess crossover in a normal pair of homologous chromosomes if crossover formation does not occur in one or two sets of unpaired chromosomes during *C. elegans* oocyte meiosis (Herman and Kari 1989; Carlton *et al.* 2006; Li *et al.* 2018). A similar increase in crossover formation between normal chromosome pairs has been observed during *Drosophila* female meiosis when chromosomal rearrangements (heterozygous inversions or translocations) are present in the cell (Sturtevant 1919; Sturtevant 1921) .

Furthermore, we examined whether the *brc-1* mutation suppressed the number of COSA-1 foci in a *him-8* and *zim-2* backgrounds. Similar to the wild-type, 97.4% of cells showed six GFP::COSA-1 foci in *brc-1* mutants ( Figure 1A, 1B). GFP::COSA-1 foci were further increased in *brc-1 ; him-8* and *brc-1 ; zim-2* double mutants compared to *him-8* and *zim-2* single mutants. A total of 62.1% and 86.5% of cells had more than six foci of GFP::COSA-1 in *brc-1 ; him-8* and *brc-1 ; zim-2* double mutants, respectively (p<0.00001; Mann-Whitney U test, comparison with each single mutant). These results suggest that BRC-1 suppresses the excess of COSA-1 foci in the *him-8* and *zim-2* mutant backgrounds.

One of the major roles of BRC-1 is to stabilize RAD-51 filaments when crossover formation is impaired (Janisiw *et al.* 2018; Li *et al.* 2018). RAD-51 levels in pachytene are elevated in *him-8* and *zim-1* mutants compared to those in the wild-type (Carlton *et al.* 2006; Li *et al.* 2018). Removal of BRC-1 in *zim-1* mutants results in a “dark zone” of RAD-51 in mid to late pachytene in which the accumulation of RAD-51 foci are suppressed (Li *et al.* 2018). To examine whether BRC-1 promotes RAD-51 filament stability in *him-8* mutants, we compared RAD-51 levels in four individual zones from the first cell of the transition zone to the end of late pachytene in the wild-type, *him-8* , and *brc-1 ; him-8* double mutants ( Figure 1C, 1D, 1E). Similar to a previous study, the levels of RAD-51 foci were elevated in all pachytene stages (zones 2, 3, and 4) in *him-8* mutants compared with the wild-type (Carlton *et al.* 2006) ( Figure 1C, 1E). The RAD-51 levels were decreased to 81% in mid-pachytene (zone 3) in *brc-1 ; him-8* double mutants compared to *him-8* single mutants (p<0.0001); however, obvious RAD-51 dark zone was not observed in *brc-1 ; him-8* double mutants. Most RAD-51 foci remain in the late pachytene stage (zone 4) in *brc-1 ; him-8* double mutants compared with *him-8* single mutants (p<0.0001). These results suggest that the contribution of BRC-1 to RAD-51 filament stability at mid-pachytene in *him-8* mutants was not as high as that in the *zim-1* mutant.

We observed an excess number of GFP::COSA-1 foci in the *him-8* and *zim-2* single mutants compared with the expected value of the five autosome pairs. These findings are consistent with those of previous studies, which demonstrated that crossover formation increases in autosomes during oogenesis in *him-8* mutants (Herman and Kari 1989; Carlton *et al.* 2006). These data suggest that interchromosomal effects occur in *him-8* and *zim-2* mutants, similar to *zim-1* mutants (Li *et al.* 2018).

The phenotypes of *brc-1 ; him-8* ( *zim-2* ) and *brc-1 ; zim-1* differed during oogenesis. The *brc-1* mutation suppresses the formation of COSA-1 foci in *zim-1* mutants (Li *et al.* 2018), whereas the *brc-1* mutation enhanced the formation of COSA-1 foci in *him-8* and *zim-2* mutants in the present study. A RAD-51 dark zone was observed in *brc-1 ; zim-1* double mutants (Li *et al.* 2018), whereas in *brc-1 ; him-8* double mutants, a slight decrease in RAD-51 levels, but no clear RAD-51 dark zone, was observed. These observations suggest that the function of BRC-1 in the formation of COSA-1 foci and the processing (removal/stabilization) of RAD-51 may be regulated differently according to the number of chromosome pairs involved in oogenesis. Furthermore, COSA-1 foci formation is differentially regulated during oogenesis and male spermatogenesis (Li *et al.* 2020). The single X chromosome condition in wild-type, *him-8* mutant *,* and *brc-1 ; him-8* double mutant males did not increase the number of COSA-1 foci compared with the hypothesized number of five COSA-1 foci (Li *et al.* 2020). In contrast to oogenesis, *brc-1 ; zim-1* males demonstrated enhanced formation of COSA-1 foci compared to *zim-1* single mutants (Li *et al.* 2020). Further research is required to explore the sex-specific regulation of crossover designs under pairing defect conditions.

## Methods

Fixation and immunostaining:

Worms expressing GFP::COSA-1 (post L4 22–24 h) were dissected using a scalpel. For dissection, 30 µL of 15 mM sodium azide solution was placed on a cover glass, followed by approximately 20 worms. After removing 15 µL of the sodium azide solution, 15 µL of 2% Paraformaldehyde (PFA) was added, mixed to a final concentration of 1% PFA and left for 5 min to fix. After removing 15 µL of the mixture, the sample was sandwiched in a slide glass. The slide was placed at –80°C for 5 min. After removing the cover glass by cracking it with a razor blade, it was fixed again in ice-cold methanol (–20°C) for 1 min. Immunostaining was performed as previously described (Saito *et al.* 2009). The primary and secondary antibodies used in this study were rabbit anti-RAD-51 antibody ( (Das *et al.* 2022), 1:3,000) and goat FITC-conjugated anti-rabbit antibody (Jackson ImmunoResearch, 1:200), respectively. The sample was then washed with phosphate-buffered saline with Tween 20 for 5 min for three times, mounted with 8 µL VECTASHIELD with 4',6-diamidino-2-phenylindole (Vector laboratories, Burlingame, California, USA), and the cover glass was shielded by nail polish.

Imaging:

Images were obtained using an all-in-one fluorescence microscope (BZ-X800; KEYENCE, Osaka, Japan) equipped with a Plan Apochromat 100x objective (NA1.45; BZ-PA100; KEYENCE). Approximately 30–40 Z-stack images of germline nuclei were captured at intervals of 0.2 µm. Green fluorescence was detected using a GFP filter (excitation, 470/40 nm; emission, 525/50 nm; dichroism, 495 nm; OP-87763; KEYENCE, Osaka, Japan). The BZ-X analysis software (BZ -H4A) and a 3D application (BZ-H4R) were used for the analysis.

## Reagents

Strains:

**Table:** 

Strain	Genotype	Available from
N2	*Caenorhabditis elegans* wild-type	CGC
TTS65	*brc-1 ( tm1145 ) III *	NBRP
TTS185	*him-8 ( tm611 ) IV *	NBRP
TTS272	*zim-2 ( tm574 ) IV *	NBRP, CGC
TTS186	*brc-1 ( tm1145 ) III; him-8 ( tm611 ) IV *	Saito lab
TTS321	*brc-1 ( tm1145 ) III; zim-2 ( tm574 ) IV *	Saito lab
AV630	*meIs8 [pie-1promoter::gfp:: cosa-1 + unc-119 (+)] II *	Villeneuve lab, CGC
TTS171	*meIs8 [pie-1promoter::gfp:: cosa-1 + unc-119 (+)] II; brc-1 ( tm1145 ) III *	Saito lab
TTS175	*meIs8 [pie-1promoter::gfp:: cosa-1 + unc-119 (+)] II; him-8 ( tm611 ) IV *	Saito lab
TTS320	*meIs8 [pie-1promoter::gfp:: cosa-1 + unc-119 (+)] II; zim-2 ( tm574 ) IV *	Saito lab
TTS174	*meIs8 [pie-1promoter::gfp:: cosa-1 + unc-119 (+)] II; brc-1 ( tm1145 ) III; him-8 ( tm611 ) IV *	Saito lab
TTS319	*meIs8 [pie-1promoter::gfp:: cosa-1 + unc-119 (+)] II; brc-1 ( tm1145 ) III; zim-2 ( tm574 ) IV *	Saito lab

After receiving the original strains, TTS65 , TTS185 , TTS186 , TTS272 , and AV630 were outcrossed six times with N2 at the Saito Laboratory.
